# Lie to Me: Shield Your Emotions from Prying Software

**DOI:** 10.3390/s22030967

**Published:** 2022-01-26

**Authors:** Alina Elena Baia, Giulio Biondi, Valentina Franzoni, Alfredo Milani, Valentina Poggioni

**Affiliations:** 1 Department of Mathematics and Computer Science, University of Florence, Viale Morgagni 67/a, 50134 Florence, Italy; alinaelena.baia@unifi.it; 2 Department of Mathematics and Computer Science, University of Perugia, Via Vanvitelli 1, 06123 Perugia, Italy; giulio.biondi@unipg.it (G.B.); or milani@unipg.it (A.M.); 3 Department of Computer Science, Hong Kong Baptist University, Kowloon Tong, Hong Kong, China

**Keywords:** emotion recognition, adversarial machine learning, privacy protection, evolutionary algorithm

## Abstract

Deep learning approaches for facial Emotion Recognition (ER) obtain high accuracy on basic models, e.g., Ekman’s models, in the specific domain of facial emotional expressions. Thus, facial tracking of users’ emotions could be easily used against the right to privacy or for manipulative purposes. As recent studies have shown that deep learning models are susceptible to adversarial examples (images intentionally modified to fool a machine learning classifier) we propose to use them to preserve users’ privacy against ER. In this paper, we present a technique for generating Emotion Adversarial Attacks (EAAs). EAAs are performed applying well-known image filters inspired from Instagram, and a multi-objective evolutionary algorithm is used to determine the per-image best filters attacking combination. Experimental results on the well-known AffectNet dataset of facial expressions show that our approach successfully attacks emotion classifiers to protect user privacy. On the other hand, the quality of the images from the human perception point of view is maintained. Several experiments with different sequences of filters are run and show that the Attack Success Rate is very high, above 90% for every test.

## 1. Introduction

Visual Emotion Recognition (ER) is one of the first Affective Computing techniques [1] that have been widely studied in computer science and artificial intelligence, based on visual features of the facial expression. Several different approaches at visual recognition obtained different grades of classifications, from face landmarks to ER using deep learning and knowledge transfer [2,3,4,5], which is currently the most common approach to facial ER. Using a Convolutional Neural Network (CNN) to detect basic emotions from an image or video frame, the resulting accuracy in the Ekman model of emotions is promising and has been implemented already [6]. Facial ER [2,6] can obtain excellent results with relatively small data sets of images, when trained on a single individual. Many are the ethical application areas in research of facial ER, e.g., to detect particular states needing an immediate medical intervention, or changes over time underlying a degenerative health condition. On the other hand, the most common applications of facial ER are prone to potentially unethical manipulation of users’ preferences. In behavior-tracking applications, the emotional reactions of a user in front of a product could produce extremely precious insights for companies, government, or political parties, prying into the user’s habits and emotional states. e.g., marketing applications in a supermarket, in front of a shop showcase, or browsing an e-commerce website [7,8]; tracking drivers’ states [9]; analyzing pieces of information in social networks [10]; analyzing news or political opinions [11]; military robot interaction [12]). For the critical nature of such information, tracking it could open a breach in personal data confidentiality, and become a potential source of manipulation bias for the user’s preferences.

The diffusion and the wide use of deep learning methods for artificial intelligence systems, thus, pose significant security and privacy issues. From the security point of view, Adversarial Attacks (AA) showed that deep learning models can be easily fooled [13,14,15,16,17,18,19,20,21,22,23,24,25] while, from a privacy point of view, it has been shown that information can be easily extracted from dataset and learned model [26,27,28]. It has also been shown that attacking methods based on adversarial samples can be used for privacy-preserving purposes [29,30,31,32,33]: in this case, data are intentionally modified to avoid unauthorized information extraction by fooling the unauthorized software.

With the increasing popularity of online social networks, privacy-preserving photo sharing has received considerable attention, and several systems have been proposed [33,34,35,36].

In this paper, we propose a technique for Emotion Adversarial Attack (EAA) to filter out the emotional features from video frames or photos of human faces to ensure the users’ freedom and protection against emotion recognizers in environments where they may be unauthorized and therefore prying.

The fooling protecting filters are built by composing and parametrizing popular image-enhancing Instagram filters: they are the result of an optimization process implemented by a nested-evolutionary algorithm [13,14,33]. Applying these protecting filters to any image, we obtain a series of other images from which information extraction is more difficult.

Since the algorithm works in a black-box scenario, it does not require any information about the model’s parameters or gradient values, as many other systems require.

The proposed algorithm combines the idea of a Multi-Objective Evolutionary (MOE) approach for adversarial attacks [13] with the *per-instance* approach presented in [33]. Compared to the MOE approach, we also introduce the use of a Full-Reference Image Quality Assessment (FR-IQA).

The *per-image* approach allows the discovery of personalized sequences of filters having different image-specific characteristics; the image assessment allows creating high quality and natural-looking adversarial privacy-preserving samples. The preferred aspect can be chosen by the user, while the image quality is controlled by the multi-objective fitness function implemented by the Structure Similarity Index (SSIM) [37].

This approach allows to overcome the main flaws of restricted attack methods that in general produce not semantically meaningful modifications that are easily detectable by software, even if they are imperceptible by human eyes [38,39,40].

Moreover, performing the attack using well-known filters widely used in social media (e.g., Instagram) makes our filter composition indistinguishable from any other filter composition extensively used every day to enhance photos and images. This approach essentially makes our attacks transparent to the human perception, still keeping their privacy-preserving emotional features.

We tested the algorithm on the AffectNet data set [41] varying the length of the sequence (3, 4 and 5 filters) obtaining attack success rates up to 96%.

## 2. Background

### 2.1. Emotion Adversarial Attacks

For an input facial image $x\in X\subset R^{d}$ and the related label *y*, let *F* be a Neural Network (NN) classifier that correctly predicts the emotional class label for the input image x:F(x)=y. An EAA attempts to modify *x* adding a δ perturbation into an adversarial image $x^{*}=x+\delta$, such as to induce *F* to make a *faulty emotion class prediction*, i.e., $F\left(x^{*}\right)\neq F\left(x\right)$.

If we consider the type of perturbation applied δ, the attacks can be classifiable as either *restricted* or *unrestricted*. If restricted, the changes applied to the original image are typically small and bounded by a $L_{p}$-norm distance, forcing the adversarial image $x^{*}$ to be as similar as possible to the initial input. On the other hand, unrestricted attacks use large unconstrained $L_{p}$-bounded perturbations manipulating the image to create adversary photorealistic instances. In this case, the intent is not to restrict the transformations on pixels but to limit the human perception that a transformation has been applied [17,42,43].

### 2.2. Image Filters

Inspired by Instagram, which offers tools to seamlessly modify images, we propose to combine multiple image filters to create custom adversarial image transformations. This approach provides plenty of styles options, ranging from subtle and warm looks to more dramatic and vivid colors effects.

As proposed in [13,14,33], we have used popular Instagram image filters such as *Clarendon, Juno, Reyes, Gingham, Lark, Hudson, Slumber, Stinson, Rise,* and *Perpetua*. Each filter has distinct properties and aspects, such as different contrast, saturation, brightness, and shadow levels. These differences allow the production of different effects that are usually composed by the users, e.g., *Rise* mixes a radial gradient with a sepia hue, while *Clarendon* brightens and highlights the image, *Juno* increases saturation, and Gingham provides a vintage appearance.

Each filter is parameterized by two values to be optimized by the algorithm: *intensity* α and *strength* *s*. The role of α is to alter the intensity of each filter component, e.g., contrast, saturation, brightness, gamma correction, edge enhancement.

The *s* parameter is used to manage the filter impact, defined as the convex interpolation between the input photo *x* and the altered image $x^{*}$:

(1) $$strength\left(x,x^{*},s\right)=\left(1.0-s\right)\cdot x+s\cdot x^{*}$$

The cases are s=0, where the image is not altered by the filter, and s=1 where the filter returns a mutated image $x^{*}$.

### 2.3. Image Quality Assessment

Image quality assessment (IQA) techniques are used to quantify the visual quality of an image by analyzing different characteristics such as aesthetics, naturalness, or distortions [44,45,46]. IQA methods are used for a variety of applications, ranging from benchmarking image processing algorithms or monitoring image quality to optimizing algorithms in the context of visual communication systems. Over the years, many different methods have been proposed. There are essentially two types of IQA methods, *subjective* and *objective*. Subjective assessment requires a human evaluation and intervention and is considered the most accurate and reliable. However, it is time-consuming, expensive, and impractical for real-time assessment applications.

Objective methods are designed to measure the visual quality of an image automatically fitting the human assessment. Using mathematical models or deep learning approaches, they result highly efficient and ideal for image-based system optimization.

Based on the availability of the reference image, objective methods can be further divided into three categories: *Full-Reference* (FR), *Reduced-Reference* (RF), and *No-Reference* (NR). FR strategies require computing the quality score by comparing the modified image with the complete reference image. RF strategies use only partial information from the reference image, such as extracted features. NR strategies, also known as blind assessments, are designed to accurately predict the image quality without using a reference image or any additional information, thus being suitable for applications where the reference image is not available.

Given the configuration of the proposed algorithm and the availability of clean reference images in our work, we use the SSIM index [37], a well-known and well-performing FR-IQA method to automatically and objectively assess the quality of adversarial samples generated by the EAA.

SSIM, introduced by Wang et al. [37], is an FR perceptual metric that quantifies the image degradations as perceived changes in the structural information. SSIM is a content-aware assessment metric, inspired by the Human Visual System (HVS), capable of extracting and identifying structural information from natural scenes (i.e., images), deeply structured with significant dependencies between spatially closed pixels. A measure that exploits the characteristics of the HVS can better match the subjectively-perceived visual quality. Capturing the change in structural information provides a good approximation of the perceived image degradation.

Structural information is defined as attributes describing objects independent from luminance and contrast. In other words, SSIM is a structural similarity measure that compares patterns of pixels intensities normalized for contrast and brightness, which variability does not alter the structures of the objects in the images. Given that different regions of an image may have different levels of contrast and luminance, the SSIM index is computed locally within a predefined 1-pixel local window, and the overall image quality is evaluated by taking the mean for the number of local windows of the image. The SSIM index is thus a multiplicative combination of three terms of comparison: luminance, contrast, and structure, computed over the image’s patches. SSIM was designed to satisfy symmetry, boundedness (i.e., where the score is bounded by an upper value equal to 1), and unique maximum property where the SSIM score is equal to 1 if and only if the two compared images are identical. In general, with a score value higher than 0.99, the images are considered to be indistinguishable (Algorithm 1).


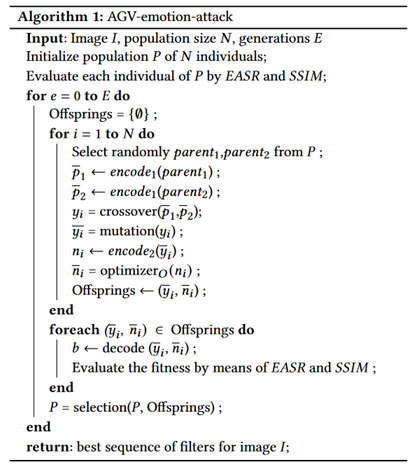


## 3. Related Works

Adversarial attacks to emotion recognition is a very recent application and just very few works are available in the literature [47,48,49]. The main difference with our work relies on the approach: white-box versus black-box. Since our algorithm works in a black-box scenario, it does not require any information about the model’s parameters or gradient values, as the other systems require. Hence, our approach can be applied against any system without having any knowledge about it. Moreover, they also differ in the way the images are modified.

In particular [49] belongs in the category of physical attacks since it realizes attacks to facial biometric systems by printing a pair of eyeglass frames.

In [47,48], a saliency map extractor is used to extract the essential expression features of the clean facial expression example and a face detector is employed to find the position of the face in the image. This information is then used to enhance and cut the gradient of the input samples computed by the optimized momentum iterative method (OMIM) with respect to the misclassification loss.

## 4. Emotion Recognition Settings

An emotion recognizer, based on MobileNetV2 [50] with transfer learning, has been designed as the target of the emotion adversarial attack image generation algorithm, to deceive the emotion classifier, while maintaining a realistic human perception quality. The emotion recognizer, a CNN, classifies a human face in the seven basic emotions of the Ekman model of seven emotions [51], *Anger*, *contempt*, *disgust*, *fear*, *happiness*, *sadness*, *surprise*, extended with an eighth *neutral* class.

### 4.1. Data Set and Data Preparation

The AffectNet [41] data set, composed of 291,651 images labeled within the eight categories of the extended Ekman model, has been used to fine-tune the classifier (see Section 4.1). AffectNet is among the most widely used data sets for ER, and provides a large amount of images to be used for ER and selected for EAA. Since the categorical distribution in AffectNet samples is unbalanced, with *happiness* and *neutral* accounting together for about 2/3 of the data set, data have been randomly sampled to optimize classes with 3500 images per emotion, for a total of 28,000 images. An 80–20% proportion has been used in randomly splitting images in each category between the training and test sets. Data augmentation has been performed to optimize the training phase, applying random horizontal flipping and horizontal/vertical shifting to the images, by a random offset in the [−15,+15] pixels range.

### 4.2. Emotion Recognizer Structure and Training

As with Transfer Learning (TL) the number of samples for NN training can be smaller than training a neural network from scratch, TL is particularly suitable in our case. TL typically allows adapting a NN (in our case, our convolutional neural network), pre-trained on a large image data set on several classes, to a network able to classify into a smaller set of possibly different categories, by using a smaller image data set and a faster training phase.

TL is based on using the pre-trained network structure and weights and replacing the last Fully-Connected (FC) classification layers with new, domain-specific layers and thus learning and adapting, i.e., *fine-tuning*, only their new weights. The idea behind this method is that different layers in a deep convolutional neural Network take into account different features; in particular, the top layers consider domain-agnostic primitive visual features, e.g., lines, and pixel color features. Deeper layers recognize more complex shapes and color distributions; the last layers in the network are responsible for assembling, by learning appropriate weights, the previously learned features into domain-specific information used for general image classification. Replacing the final layers allows the network to keep its low-level features recognition ability, saving training time, and adapting it to a new domain by re-training the new layers only.

In order to build the emotion recognizer by transfer learning, the MobileNetV2 [50] network was chosen, for its relatively small number of parameters, i.e., size. MobileNetV2 is pre-trained on the ImageNet data set [52,53] and it is able to classify images into 1000 categories. It is relevant to notice that different neural networks can have different performance on different data sets, and chosing a commonly-used data set as AffectNet, and a NN with a low number of parameters enhances the experiment clarity, still allowing future comparison with other approaches, datasets and networks. In the emotion recognizer deep network structure, the last MobileNetV2 fully connected classification layer is replaced with a FC layer of size 128 followed by a 0.5 dropout layer and a final fully connected layer of size 8 for the emotion classes, where the FC layers activation functions are, respectively, ReLU and Softmax.

Cross-validation in the CNN training is used to find the best set of hyperparameters obtained by the optimizer for given data. We specified the mini-batch size to 10, and validation data are shuffled at the beginning of each epoch. An epoch is a full training cycle on the entire training data set (i.e., 80% for our hold-out split).

During the training of the ER, starting with the initial pre-trained MobileNetV2 weights, we used the Stochastic Gradient Descent with Momentum (SGDM) as an optimizer, a *piecewise decay* optimizer policy, and a variable learning rate from an initial value of $1\times 10^{-3}$, halving every 10 epochs on a total of 80.

## 5. Algorithm for Adversarial Attacks

The algorithm used to produce the attacks is implemented by mixing the idea of the MOE approach proposed in [13] and the *per-instance* approach proposed in [33]. We decided to use the MOE approach, which allows maximizing the method effectiveness and minimizing the image distortion. With a simpler optimization criterion, e.g., using the attack success rate only, the images could be excessively modified, creating an unnatural look. The description of the algorithm is given in Algorithm 1.

The optimization method consists of two nested evolutionary algorithms: an *outer algorithm*, using a generative adversarial approach based on a genetic algorithm, in charge of finding the sequence of filters to use, and an *inner algorithm*, based on Evolution Strategy (ES), used to choose the values of parameters. Given a set $S=\{f_{1},f_{2},\cdots f_{m}\}$ of *m* image filters, the outer algorithm genotype (with length *l*) is encoded as a list of filters, while the inner algorithm genotype is represented by a list containing the parameters for each selected filter.

### 5.1. Outer Algorithm

The outer-algorithm optimization is performed by a genetic algorithm: a population of *N* candidates is iteratively evolved. The candidates are randomly chosen to breed a new generation by the crossover and mutation procedures, where the candidates are evaluated on their fitness. At the end of each iteration, the best candidates are selected for the next generation:

**Initial population:** Generated by randomly selecting *l* filters from the *S* available set, and their parameters are initialized to 1.
**Crossover:** We use a one-point crossover to generate new off-springs (i.e., children) from random members. Each child is assured of inheriting genetic information from both parents.
**Mutation:** A filter is replaced with another, on a probability of mutation. The new filter is initialized with random parameters, assuring their complete mutation.
**Selection:** At each iteration, the N best individuals are chosen from the set of 2N candidates (i.e., parents and offsprings), according to their fitness. The same process is repeated until the algorithm spends the fixed amount of generations. The selection is implemented as a multi-objective evolutionary problem based on two criteria: Attack Success Rate (ASR) and image quality (evaluated by SSIM). The addition of the image quality assessment in the population evaluation phase gives the algorithm the capabilities to create high-quality and natural-looking adversarial examples. Given *F* a target *facial emotion recognizer*, $x_{i}$ an original facial image, $x_{i}^{*}$ derived from $x_{i}$ by applying a sequence of filters, the fitness function is evaluated by the following:
  (2) $$F\left(x_{i},x_{i}^{*}\right)=\left\{1.0-EASR_{i}\left(x_{i},x_{i}^{*}\right),1-SSIM_{i}\left(x_{i},x_{i}^{*}\right)\right\},$$
  where $EASR_{i}$ is the emotion adversarial attack success Rate obtained by classifying the modified image $x_{i}^{*}$ with the target emotion classifier:
  (3) $$EASR_{i}=\left\{\begin{array}{c} 1,\;if\;F\left(x_{i}\right)\neq F\left(x_{i}^{*}\right)\\ 0,\;otherwise, \end{array}\right.$$
  and $SSIM_{i}$ represents the image quality score that controls the amount of the applied perturbation described in Section 2.3.

### 5.2. Inner Algorithm

The inner algorithm is committed to the optimization of parameters, accomplished by (1,λ) evolution strategy with λ=5. A search distribution is iteratively updated by ES, following the gradient towards increased expected fitness. For each list of parameters, we compute *N* candidates through a perturbation of the original individuals. The gradient is estimated to better solutions comparing the fitness values of the *N* candidates. The gradient is then used to replace the previous individual. The entire process is repeated until meeting a stopping criterion.

## 6. Experiments and Discussion

### 6.1. Experimental Setup

The proposed algorithm has been evaluated by attacking the *Emotion Recognizer* described in Section 4, which is based on the MobileNetV2 neural network, adapted, for the emotion recognition task, using transfer learning techniques and the well-known facial expression dataset AffectNet.

The experiments have been run on a subset of the correctly classified image from the validation set. 10 images have been randomly selected, for each class for a total of 80 images. Moreover, three different experimental configurations have been defined, based on the number of filters applied to the input image: we used sequences of length equal to 3, 4, and 5. The filters’ parameters *intensity* α and *strength* *s* are initialized with default values equal to 1.

Extending the concept of transfer learning, we decided to use hyperparameters that have been found for other problems [13,33]. As the results presented in Section 6.3 demonstrate, this proved to be a successful strategy as it allowed us to save time and computational effort without loss in protection effectiveness. Thus, we have chosen the following setup: for the outer algorithm, a population size = 10, mutation probability = 0.5, and 10 generations; the population size of the inner algorithm has been fixed to 5, and the number of generations to 3.

### 6.2. Evaluation

The system effectiveness is evaluated by the overall emotion attack success rate defined as:

(4) $$EASR\left(X,X^{*}\right)=\frac{1}{n}\sum_{i=0}^{n}F\left(x_{i}\right)\neq F\left(x_{i}^{*}\right),$$

where *n* is the dataset size, and $x_{i}$ and $x_{i}^{*}$ are images from dataset *X* and the corresponding modified dataset $X^{*}$. We chose this measure as the standard evaluation measure for adversarial attacks to measure the percentage of images in the dataset for which the emotion recognizer fails the classification.

### 6.3. Results and Generated Images

The experimental results show that the algorithm can reach a high attack success rate EASR. More specifically, it achieves 91.25%, 93.75%, and 96.25% when using 3, 4, and 5 filters, respectively.

**Figure 1:**
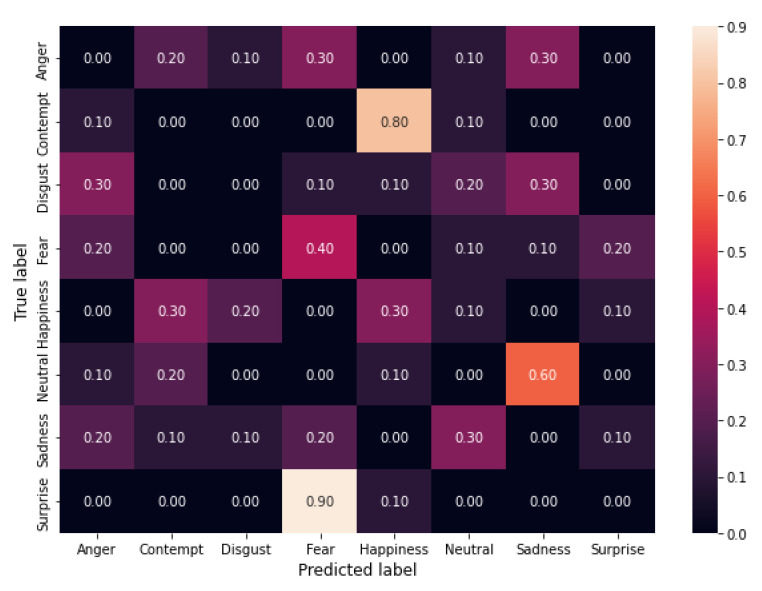
Confusion matrix from the results of the attack with three filters.

**Figure 2:**
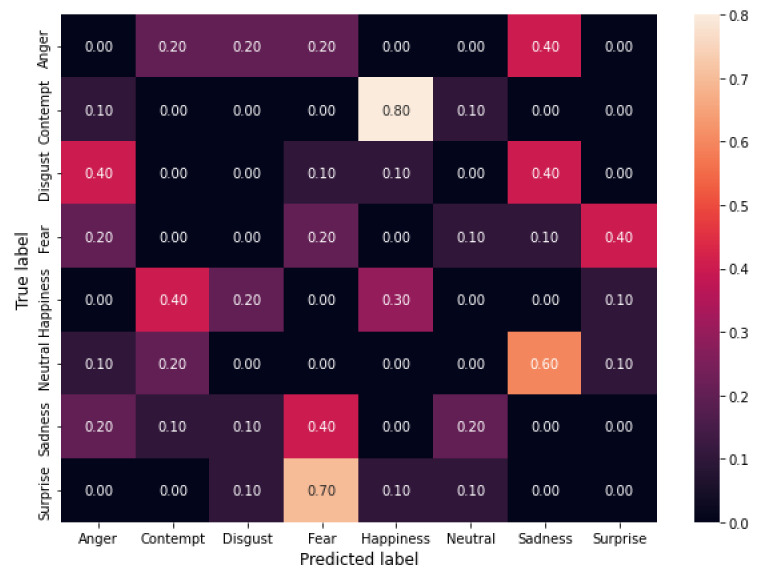
Confusion matrix from the results of the attack with four filters.

**Figure 3:**
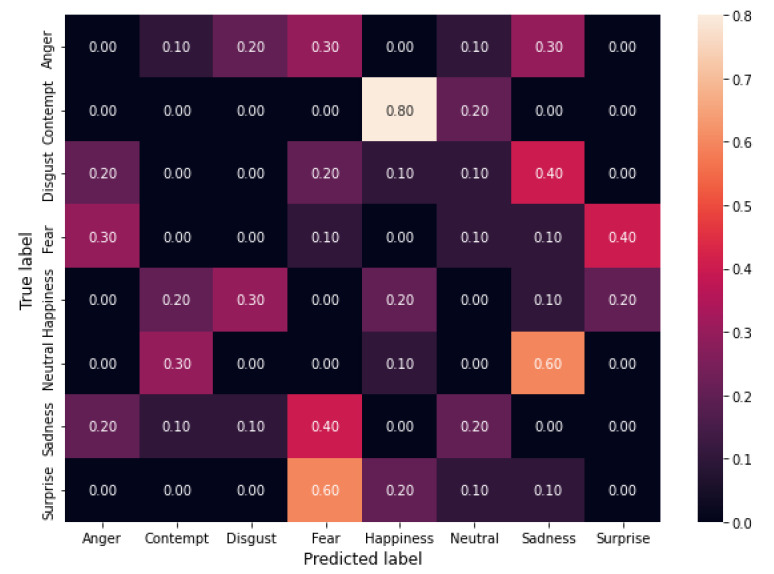
Confusion matrix from the results of the attack with five filters.

In Figure 1, Figure 2 and Figure 3 the confusion matrices obtained by the three experiments are shown.

Confusion matrices allow evaluating the error distribution among classes. We can note that an increase of the length of filter sequences corresponds to increasing EASR and that the only classes that maintain some correct classifications are *fear* and *happiness* while all the others show an EASR of 100%. We ca also note that for the classes *Contempt*, *Neutral* and *Surprise* we obtained a shift (a number of errors greater than 50%) towards another class *Contempt* →*Happiness*, *Neutral*→*Sadness* and *Surprise*→ *Fear*, while for the other five classes the errors are quite-uniformly distributed among the other classes.

We have also studied the impact of the filters on the images. In Table 1, some examples are shown. For each original image in the first column, the results obtained for sequences of 3, 4, and 5 filters are reported. We can note that the algorithm can produce natural-looking and artifacts free adversarial samples. This effect is due to the uniform application of the filters across the entire image, and the controlled perturbations through the SSIM index.

**Figure 4:**
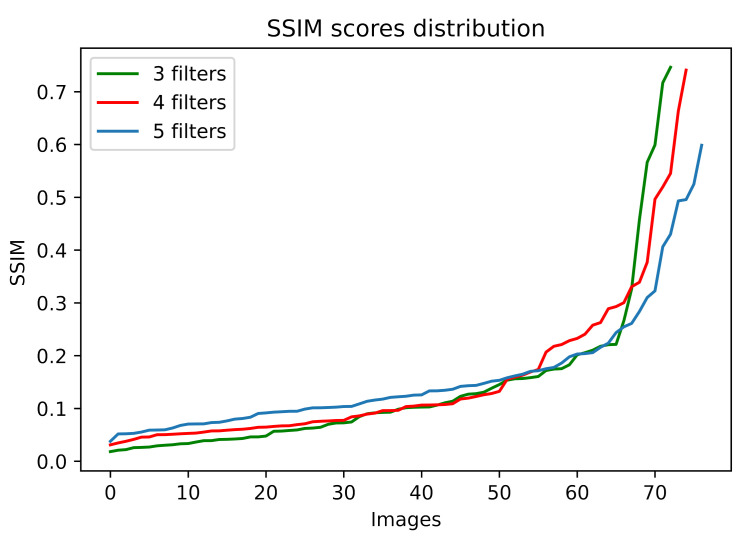
SSIM values distributions for the attacking images produced by sequences of 3, 4 and 5 filters.

Moreover, to have a global view of the impact in terms of SSIM index, we have analyzed the values of the index for all the attacking images. In Figure 4, the distributions of the SSIM values for the images produced by sequences of 3, 4 and 5 filters are shown. We can observe that, for most of the images, the scores are remarkably low, and only for very few cases, they reach values above 0.3.

We can also observe no significant differences among the three versions: users can choose according to their necessities, preferring a less/more modified image at the expense of the effectiveness of the protection.

## 7. Conclusions and Future Work

In a continuously evolving AI world, the most common applications of facial emotion recognition are prone to induce potentially unethical manipulation bias for the user’s preferences. From the security point of view, deep learning models can be easily fooled by adversarial attacks, with data intentionally modified to avoid unauthorized information extraction by software. We show that combining a multi-objective evolutionary approach for AA with a per-instance approach allows the discovery of personalized sequences of filters having different image-specific characteristics, which can filter out the emotional features for the prying software. Composing and parametrizing popular image-enhancing Instagram filters on video frames or photos of human faces, the sequence is optimized by a nested-evolutionary algorithm, not requiring any information about the model’s parameters or gradient values. Applying these protecting filters to any image we obtain a series of other images from which emotional information extraction is more difficult, while the transformation is transparent to the human perception, still keeping a natural look and their privacy-preserving emotional features. After a series of preliminary tests to have the best trade-off between computation efforts, time, and attacking effectiveness, achieving 91.25%, 93.75%, and 96.25% when using 3, 4, and 5 filters, respectively. The only classes that maintain some correct classifications are *fear* and *happiness*, all the others show an emotion adversarial attack success rate of 100%. Moreover, the algorithm can produce natural-looking and artifacts-free adversarial samples by applying the filters across the entire image and controlling perturbations through the structure similarity index.

## Figures and Tables

**Table 1:** Examples of adversarial samples: the first column reports the original image and original classification. Columns 2–4 show the adversarial images with their classification. We can notice how the adversarial attack changes the automated emotion recognition without disrupting the image appearance.

Original	3 filters	4 filters	5 filters
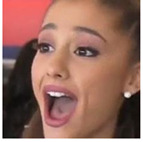	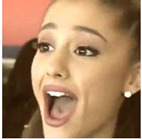	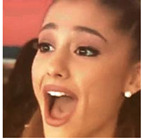	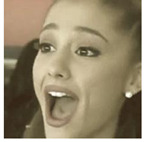
**surprise**	fear	fear	fear
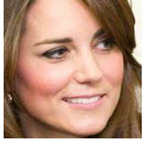	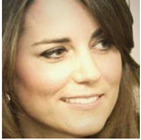	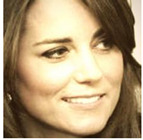	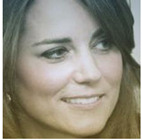
**happiness**	contempt	contempt	disgust
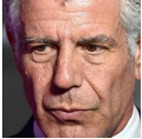	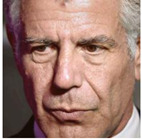	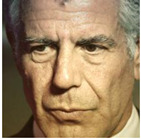	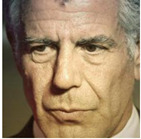
**anger**	sadness	sadness	sadness
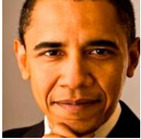	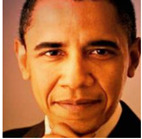	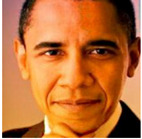	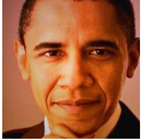
**happiness**	contempt	contempt	contempt

## Data Availability

Source code is publicly available at https://github.com/Ellyuca/AGV-Project, accessed on 30 December 2021.
